# Supplementary material for: Heat adaptation of phage T7 under an extended genetic code
Source: Virus Evol. 2021 Dec 1;7(2):veab100. doi: 10.1093/ve/veab100 (PMC8923235; doi:10.1093/ve/veab100)
Supplement: veab100_Supp [file veab100_supp.zip › Sequencing_Analysis.zip!/Sequencing_Analysis/A1/evidence/RA_39.html]

BRESEQ :: Evidence


| Read alignment evidence... | | | | | | | | | | | |
| --- | --- | --- | --- | --- | --- | --- | --- | --- | --- | --- | --- |
|  | seq id | position | | ref | new | freq | score (cons/poly) | reads | annotation | genes | product |
| \* | NC\_001604.1 | 33,069 | 0 | G | T | 26.2% | 7113.9 / inf | 3808 | intergenic (–/–) | *–/–* | –/– |
| Reads supporting (aligned to +/- strand):  **ref** base G (1566/1236);  **new** base T (578/415);  **total** (2151/1657) | | | | | | | | | | | |
| Fisher's exact test for biased strand distribution *p*-value = 2.19e-01 | | | | | | | | | | | |
| Kolmogorov-Smirnov test that lower quality scores support variant *p*-value = 5.59e-01 | | | | | | | | | | | |

Only 100 of 3837 total aligned reads displayed.  

`GATGGTAGAGAAGTACGCTATGGATAAGGCTTATGGTATCTCCCACTCAGACCAGTTCACCAACAGTTCCATAATAGAAGAGAACATTGAGGGCTTAGTAGGTATCGAGAATAACTCATTCCTTGAGGCACGTAACTTGTTTGATTCGGACCTATCCATCACTATGCCAGACGGACAGCAATTCTCAGTGAATGACCTAAGGGACTTCGATATGTTCCGCATCATGCCAGCGTATGACCGCCGTGTCAATGGTGACATCGCCATCATGGGGTCTACTGGTAAAACCACTAAGGAACTTAAGGATGAGATTTTGGCTCTCAAAGCGAAAGCTGAGGGAGACGGTAAGAAGACTGGCGAGGTACATGCTTTAATGGATACCGTTAAGATTCTTACTGGTCGTGCTAGACGCAATCAGGACACTGTGTGGGAAACCTCACTGCGTGCCATCAATGACCTAGGGTTCTTCGCTAAGAACGCCTACATGGGTGCTCAGAACATTACGGAGATTGCTGGGATGATTGTCACTGGTAACGTTCGTGCTCTAGGGCATGGTATCCCAATTCTGCGTGATACACTCTACAAGTCTAAACCAGTTTCAGCT  >  NC_001604.1/32769‑33369`  

                                                                                                                                                                                                                                                                                                            `|`                                                                                                                                                                                                                                                                                                              
    `gTAGAGAAGTACGCTATGGATAAGGCTTATGGTATCTCCCACTCAGACCAGTTCACCAACAGTTCCATAATAGAAGAGAACATTTAGGGCTTAGTAGGTATCGAGAATAACTCATTCCTTGAGGCACGTAACTTGTTTGATTCGGACCTATCCATCAC‑ATGCCAGACGGACAGCAATTCTCAGTGAATGACCTAAGGGACTTCGATATGTTCCGCATCATGCCAGCGTATGACCGCCGTGTCAATGGTGACATCGCCATCATGGGGTCTACTGGTAAAACCACTAAGGAACTTAAGGATga                                                                                                                                                                                                                                                                                                         >  1:92165/1‑301 (MQ=255)`  
    `gTAGAGAAGTACGCTATGGATAAGGCTTATGGTATCTCCCACTCAGACCAGTTAACCAACAGTTCCATAATAGAAGAGAACATTGAGGGCTTAGTAGGTATCGAGAATAACTCATTCCTTGAGGCACGTAACTTGTTTGATTCGGACCTATCCATCACTATGCCAGACGGACAGCAATTCTCAGTGAATGACCTAAGGGACTTCGATATGTTCCGCATCATGCCAGCGTATGGCCGCCGTGTCAATGGTGACATCGCCATCATGGGGTCTACTGGTCAAACCACTAAGGCACTTAAGGAtt                                                                                                                                                                                                                                                                                                          >  1:418555/1‑300 (MQ=255)`  
        `agaAGTAAGCTATGGATATGGCTTATGGTATCTCCCACTTATACCAGTTAACCAACAGTTCCATAATAGAGGAGAACAATGAGGGCTTAGTAGAGAACGAGAATAACTCATTCCTTGAGGGACGTAACTTGTTTGAGTCGGACCTATTAAGCTATAGGCCAGACGGACAGCAATTCTCAGAGAATGACCTATGGGATATCGATATGTTCCGCATCATGCCAGCGTATAACCGCCGTGTCAATGGTGACATCGCCATCTTGGGGTCTACTGGTAAAACCACTAAGGAACTTAAGGATGAGAt                                                                                                                                                                                                                                                                                                      <  1:200526/301‑1 (MQ=255)`  
               `cGCTATTGATAAGGCTTATGGTATCTCCCACTCAGACCAGTTCACCAACAGTTCCATAATAGAAGAGAACATTGAGGGCTTAGTAGGTATCGAGAATAACTCATTCCTTGAGGCACGTAACTTGTTTGATTCGGACCTATCCATCACTATGCCAGACGGACAGCAATTCTCAGTGAATGACCTAAGGGACTTCGATATGTTCCGCATCATGCCAGCGTATGACCGCCGTGTCCATGGTTACATCGCCATCATGGGGTCTACTGGTAAAACCACTAAGGAACTTAAGGATGAGCTTTTGGct                                                                                                                                                                                                                                                                                               >  1:65333/1‑301 (MQ=255)`  
                   `aTGGATAAGGCTTATGGTATCTCCCACTCAGACCAGTTCACCAACAGTTCCATAATAGAAGAGAACATTGAGGGCTTAGTAGGTATCGAGAATAACTCATTCCTTGAGGCACGTAACTTGTTTGATTCGGACCTATCCATCACTATGCCAGACGGACAGCAATTCTCAGTGAATGACCTAAGGGACTTCGATATGTTCCGCATCATGCCAGCGTATGACCGCCGTGTCAATGGTGACATCGCCATCATGGGGTCTACTGGTAAAACCACTAAGGAACTTAAGGATGAGATTTTGGCTCTCa                                                                                                                                                                                                                                                                                           >  1:468314/1‑301 (MQ=255)`  
                           `ggCTTATGGTATCTCCCACTCAGACCAGTTCACCAACAGTTCCATAATAGAAGAGAACATTGAGGGCTTAGTAGGTATCGAGAATAACTCATTCCTTGAGGCACGTAACTTGTTTGATTCGGACCTATCCATCACTATGCCAGACGGACAGCAATTCTCAGTGAATGACCTAAGGGACTTCGATATGTTCCGCATCATGCCAGCGTATGACCGCCGTGTCAATGGTTACATCGCCATCATGGGGTCTACTGGTAAAACCCCTAAGGAACTTAAGGATGAGATTTTGGCTCTCAAAGCGaaa                                                                                                                                                                                                                                                                                   >  1:324014/1‑301 (MQ=255)`  
                           `ggCTTATGGTATCTCCCACTCAGACCAGTTCACCAACAGTTCCATAATAGAAGAGAACATTGAGGGCTTAGTAGGTATCGAGAATAACTCATTCCTTGAGGCACGTAACTTGTTTGATTCGGACCTATCCATCACTATGCCAGACGGACAGCAATTATCAGTGAATGACCTAAGGGACTTCGATATGTTCCGCATCATGCCAGCGTATGACCGCCGTGTCAATGGTGACATCGCAAGCATGGGGTCTACTGATAAAAACACTAAGGAACTTAAGGATGAAGTTTTGGCTCACAAAGAGaaa                                                                                                                                                                                                                                                                                   >  1:352349/1‑301 (MQ=255)`  
                           `ggCTTATGGTATCTCCCACTCAGACCAGTTCACCAACAGTTCCATAATAGAAGAGAACATTGAGGGCTTAGTAGGTATAGAGAATAACTCATTCCTTGAGGCACGTAACTTGTTTGATTCGGACCTATCCATCACTATGCCAGACGGACAGCAATTCTCAGTGAATGACCTAAGGGACTTCGATATGTTCCGCATCATGCCAGCGTATGACCGCCGTGTCAATGGTGACATCGCCATCATGGGGTCTACTGGTAAAACCACTAAGGAACTTAAGGATGAGATTTTGGCTCTCAAAGCGaaa                                                                                                                                                                                                                                                                                   >  1:397588/1‑301 (MQ=255)`  
                                `aTGGTATCTCCCACTCAGACCAGTTCACCAACAGTTCCATAATAGAAGAGAACATTGAGGGCTTAGTAGGTATCGAGAATAACTCATTCCTTGAGTCACGTAACTTGTTTGATTCGGACCTATCCATCACTATGCCAGACGGACAGCAATTCTCAGTGAATGACCTAAGGGACTTCGATATGTTCCGCATCATGCCAGCGTATGAGCGCCGTGTCAATGGTTACATCGCCATCATGGGGTCTACTGGTAAAACCACTAAGGAACTTAAGGATGAGATTTTGGCTCTCAAAGCGAAAGCTGa                                                                                                                                                                                                                                                                              >  1:122016/1‑301 (MQ=255)`  
                                  `ggTATCTCCCACTCAGACCAGTTCACCAACAGTTCCATAATAGAAGAGAACATTGAGGGCTTAGTAAGTATCGAGACTAACTCATTCCTTGAGGCACGTAACTTGTTTGATTCGGACCTATCCATCACTATGCCCGACGGGCAGCAATTCTCAGTGAATGACCTAAGGGACTTCGATATGTTCCGCATCATGCCAGCGTATGACCGCCGTGTCAATGGTTACATCGCCATCATGGGGTCTACTGGTAAACCCACTACGGCACTTAAGGCTTAGCTTTTGGCTCTCAAAGCGAAAGCttatg                                                                                                                                                                                                                                                                            >  1:28322/1‑297 (MQ=255)`  
                                     `atatcgcagtCAGACCAGTTCACCAACAGTTTTATAATAGAAGAGAACATTGAGGGCGTAGTAGGTATCGAGAATAACTCATTCATTGAGGCACGTAACTTGTTTGATTCGGACCTATCCAGCACGATGCCAGCCGGACAGCAATTCTCAGTGAATGACCTAAGGGACTTCGATATGTTCCGAATCATGCCAGCGTATGACCGCCGTGTCAATGGTTACATCGCTATCATGGGGTCTACTGGTAAAACCACTAAGGAACTTAAGGATGAGATTATGGCTCTAAAAGCGAAAGCTGAGGgag                                                                                                                                                                                                                                                                         <  1:387423/292‑1 (MQ=255)`  
                                          `ccACTCAGACCAGTTCACCAACAGTTCCATAATAGAAGAGAACATTGAGGGCTTAGTAGGTATCGAGAATAACTCATTCCTTGAGGCACGTAACTTGTTTGATTCGGACCTATCCATCACTATGCCAGACGGACAGCAATTCTCAGTGAATGACCTAAGGGACTTCGATATGTTCCGCATCATGCCAGCGTATGACCGCCGTGTCAATGGTGACATCGCCATCATGGGGTCTACTGGTAAAACCACTAAGGAACTTAATGATGAGATTTTGGCTCTCAAAGCGAAAGCTGAGGGAGACGGt                                                                                                                                                                                                                                                                    <  1:145580/301‑1 (MQ=255)`  
                                            `aCTCAGACCAGTTCACCAACAGTTCCATAATAGAAGAGAACATTGAGGGCTTAGTAGGTATCGAGAATAACTCATTCCTTGAGGCACGTAACTTGTTTGATTCGGACCTATCCATCACTATGCCAGACGGACAGCAATTCTCAGTGAATGACCTAAGGGACTTCGATATGTTCCGCATCATGCCAGCGTATGACCGCCGTGTCAATGGTGACATCGCCATCATGGGGTCTACTGGTAAAACCACTAAGGAACTTAAGGATGAGATTTTGGCTCTCAAAGCGAAAGCTGAGGGAGACGTTaa                                                                                                                                                                                                                                                                  >  1:50627/1‑301 (MQ=255)`  
                                                  `aCCAGTACACCAACAGTTCCATAATAGAAGAGAACATTGAGGGCTTAGTAGGAATCTAGAATAACGCATTCCTTGAGGCACGTAACTTGTTTGATTCGGACCTAACCATCACTATGCCAGACGGACAGCAATTCTCAGTGAATGACCTAAGGGACTTCGATATGTTCCGCATCATGCCAGCGTATGACCGCCGTGTAAATGGTTACATCGCCATCATGGGGTCTACTGGTAAAACCACTAAGGAACTTAAGGATGAGATTTTGGCTCTCAAAGCGAAAGCTGAGTGAGACGGTAAGAAGAc                                                                                                                                                                                                                                                            <  1:52020/301‑1 (MQ=255)`  
                                                   `ccAGTTCACCAACAGTTCCATAATAGAAGAGAACATTGAGGGCTTAGTAGGTATCGAGAATAACTCATTCCTTGAGGCACGTAACTTGTTTGATTCGGACCTATCCATCACTATGCCAGACGGACAGCAATTCTCAGTGAATGACCTAAGGGACTTCGATCTGTTCCGCATCATGCCAGCGTATGACCGCCGTGTCAATGGTGACATCGCCATCATGGGGTCTACTGGTAAAACCACTAAGGCACTTAATTATGAGATTTTTGCTCTCACAGCGAACGCTGAGGGAGACGGTAAGAAGACt                                                                                                                                                                                                                                                           >  1:347480/1‑301 (MQ=255)`  
                                                      `gTTCACCAACAGTTCCATAATAGAAGAGAACATTGAGGGCTTAGTAGGTATCGAGAATAACTCATTCCTTGAGGCACGTAACTTGTTTGATTCGGACCTATCCATCACTATGCCAGACGGACAGCAATTCTCAGTGAATGACCTAAGAGACTTCGATATGTTCCGCATCATGCCAGCGTATGACCGCCGTGTCAATGGTTACATCGCCATCATGGGGTCTACTGGTAAAACCACTACGGAACTTAAGGATGAGATTTTGGCTCTCACAGCGAAAGCTGAGTGAGACTGTAAGAAGACTGGc                                                                                                                                                                                                                                                        >  1:476678/1‑301 (MQ=255)`  
                                                      `gTTCACCAACAGTTCCATAATAGAAGAGAACATTGAGGGCTTAGTAGGTATCGAGAATAACTCATTCCTTGAGGCACGTAACTTGTTTGATTCGGACCTATCCATCACTATGCCAGACGGACAGCAAATCTCAGTGAATGACCTAAGGGACTTCGATATGTTCAGCATCATACCAGCGTATAACCGCCGTGTCACTGGTGACATCGCCATCATGGGGTCTACTGGTAAAACCACTAAGGAACTTAATGATTAACTTTTGGCTCTCATAGCAAAAGCTGAAGGAGACGGGAAAAAGACTTGc                                                                                                                                                                                                                                                        >  1:55673/1‑301 (MQ=255)`  
                                                            `gacaggTTCCATAATAGAAGAGAACATGGAGGGCTTAGTAGGTATCGAGAATAACTCATTCCTTGAGGCACGTAACTTGTTTGAGTCGGACCTATCCATCACTATGCCAGACGGACAGCAATTCTCAGTGAATGACATAAGGGACTTCGATATGTTCCGCATCATGCCAGCGTATGACCGCCGTGTCAATGGTGACATCGCCATCATGGGGTCTACTGGTAAAACCACTAAGGAACTTAATGATGAGATTTTGGCTCTCAAAGCGAAAGCTGAGGGAGACGGTAAGAAGACTGGCGAGGTa                                                                                                                                                                                                                                                  <  1:373628/296‑1 (MQ=255)`  
                                                             `aaCAGTTCAATAAAAGAAGAGAAAATTGAGGGCTTAGTAGGTATCGAGAATAACTCATTCCTGGAGGCACGTAACTTGTTTGATTCGGACCTATCCAGCACTATGCCAGACGGACAGCAATTCTCAGTGAATGACCTAAGGGACTTCGATATGTTCCGCATCATGCCAGCGTATGACCGCCGTGTCAATGGTTACATCGCAATCATGGGGTCTACTGGTAAAACCACTAAGGAACTTAAGGATGAGATTTTGGCTCTCAAAGCGAAAGCTGAGGGAGACGGTAAGAAGACTGGCGAGGTAc                                                                                                                                                                                                                                                 <  1:256416/301‑1 (MQ=255)`  
                                                               `cAGTTCCATAATAGAAGAGAACATTGAGGGCTTAGTAGGTATCGAGAATAACTCATTCCTTGAGGCACGTAACTTGTTTGATTCGGACCTATCCATCACTATGCCAGACGGACAGCAATTCTCAGTGAATGACCTAAGGGACTTCGATATGTTCCGCATCATGCCAGCGTATGACCGCCGTGTCAATGGTTACATCGCCATCATGGGGTCTACTGGTAAAACCACTAAGGAACTTAAGGATGAGATTTTGGCTCTCAAAGCGAAAGCTGAGGGAGACGGTAAGAAGACTGGCGAGGTACAt                                                                                                                                                                                                                                               >  1:481986/1‑301 (MQ=255)`  
                                                                 `gTTCCATAATAGAAGAGAACATTGAGGGCTTAGTAGGTATCGAGAATAACTCATTCCTTGAGGCACGTAACTTGTTTGATTCGGACCTATCCATCACTATGCCAGACGGACAGCAATTCTCAGTGAATGACCTAAGGGACTTCGATATGTTCCGCATCATGCCAGCGTATGACAGCCGTGTCAATGGTTACATCGCCATCATGGGGTCTACTGGTAAAACCACTAAGGAACTTAAGGATGAGATTTTGGCTCTCAAAGCGAAAGCTGAGGGAGACGGTAAGAAGAATGGCGAGGTAAATGc                                                                                                                                                                                                                                             >  1:33244/1‑301 (MQ=255)`  
                                                                  `ttCCATAATAGAAGAGAACATTGAGGGCTTAGTAGGTATCGAGAATAACTCATTCCTTGAGGCACGTAACTTGTTTTATTCGGACCTATCCATCACTATGCCAGACGGACAGCAATTCTCAGTGAATGACCTAAGGGACTTCGATATGTTCCGCATCATGCCAGCGTATGACCGCCGTGTCAATGGTGACATCGCCATCATGTGGTCTACTGGTAAAACCACTAAGGAACTTAATGATGAGATTTTGGCTCTCAAAGCGAAAGCTGAGGGAGATGGTAAGAAGACTGGCGAGGTACAGGCt                                                                                                                                                                                                                                            <  1:472191/301‑1 (MQ=255)`  
                                                                       `taataGAAGAGAACATTGAGGGCTTAGTAGGTATCGAGAATAACTCATTCCTTGAGGCACGTAACTTGTTTGATTCGGACCTATCCATCACTATGCCAGACGGACAGCAATTCTCAGTGAATGACCTAAGGGACTTCGATATGTTCCGCATCATGCCAGCGTATGACCGCCGTGTCAATGGTTACATCGCCATCATGGGGTCTACTGGTAAAACCACTAAGGAACTTAAGGATGAGATTTTGGCTCTCAAATCGAAAGCTGAGGGAGACGGTAAGAAGACTGGCGAGGTACATGCTTTAAt                                                                                                                                                                                                                                       >  1:171900/1‑301 (MQ=255)`  
                                                                       `taataGAAGAGAACATTGAGGGCTTAGTAGGTATCGAGAATAACTCATTCCTTGAGGCACGTAACTTGTTTGATTCGGACCTATCCATCACTATGCCAGACGGACAGCAATTCTCAGTGAATGACCTAAGGGACTTCGATATGTTCCGCATCATGCCAGAGTATTACCGCCGTGTCAATGGTTACATCGCCATCATGGGGTCTACTGGTAAAACCACTAAGGAACTTAAGAATGAGATTTTGGCTCTCAAAGCGAAAGCTGAGGGAGACGGTAAGCAGACTGGCGAGGGACATGCTTTAAt                                                                                                                                                                                                                                       >  1:158476/1‑301 (MQ=255)`  
                                                                         `ataGAAGAGAACATTGAGGGCTTAGTAGGTATCGAGAATAACTCATTCCTTGAGGCACGTAACTTGTTTGATTCGGACCTATCCATCACTATGCCAGACGGACAGCAATTCTCAGTGAATGACCTAAGGGACTTCGATATGTTCCGCATCATGCCAGCGTATGACCGCCGTGTCAATGGTTACATCGCCATCATGGGGTCTACTGGTAAAACCACTAAGGAACTTAAGGATGAGATTTTGGCTCTCAAAGCGAAAGCTGAGGGAGACGGTAAGAAGACTGGCGAGGTACATGCTTTAATgg                                                                                                                                                                                                                                     <  1:319376/301‑1 (MQ=255)`  
                                                                         `aaataagaGAACATTGTGGGCTTAGTATGGATTGAGAATAACTCATTCCTTGCGGAACGTAACTTGTTTGATTCGGACCTATCCATCACTACGCCCGACGGACAGCAATTCTCAGTGACTGACCTAAGGGACTTCGATATGTTCCGCATCATGCCAGCGTATGACCGCCTTGTCAATGGTGACATCGCCATCATGGGGTCTACTGGTAAAACCACTAAGGAACTTAAGGATGAGATTTTGGCTCTCAAAGCGAAAGCTGAGGGAGACGGTAAGAAGACTGGCGAGGTACATGCTTTAATgg                                                                                                                                                                                                                                     <  1:100300/297‑1 (MQ=255)`  
                                                                              `agagAACATTGAGGGCTTAGTAGGTATCGAGAATAACTCATTCCTTGAGGCACGTAACTTGTTTGATTCGGACCTATCCATCACTATGCCAGACGGACAGCAATTCTCAGTGAATGACCTAAGGGACTTCGATATGTTCCGCATCATGCCAGCGTATGACCGCCGTGTCAATGGTTACATCGCCATCATGGGGTCTACTGGTAAAACCACTAAGGAACTTAAGGATGAGATTTTGGCTCTCAAAGCGAAAGCTGAGGGAGACGGTAAGAAGACTGGCGAGGTACATGCTTTAATGGATAcc                                                                                                                                                                                                                                >  1:204385/1‑301 (MQ=255)`  
                                                                                   `aCATTGAGGGCTTAGTAGGTATCGAGAATAACTCATTCCTTGAGGCACGTAACTTGTTTGATTCGGACCTATCCATCACTATGCCAGACGGACAGCAATTCTCAGTGAATGACCTAAGGGACTTCGATATGTTCCGCATCATGCCAGCGTATGACCGCCGTGTCAATGGTGACATCGCCATCATGGGGTCTACTGGTAAAACCACTAAGGAACTTAAGGATGAGATTTTGGCTCTCAAAGCGAAAGCTGAGGGAGACGGTAAGAAGACTGGCGAGGTACATGCTTTAATGGATACCGTTaa                                                                                                                                                                                                                           >  1:410493/1‑301 (MQ=255)`  
                                                                                    `cATTGAGGGCTTAGTAGGTATCGAGAATAACTCATTCCTTGAGGCACGTAACTTGTTTGATTCGGACCTATCCATCACTATGCCAGACGGACAGCAATTCTCAGTGAATGACCTAAGGGACTTCGATATGTTCCGCATCATGCCAGCGTATGACCGCCGTGTCAATGGTTACATCGCCATCATGGGGTCTACTGGTAAAACCACTAAGGAACTTAAGGATGAGATTTTGGCTCTCAAAGCGAAAGCTGAGGGAGACGGTAAGAAGACTGGCGAGGTACATGCTTTAATGGTTACCGTTAAg                                                                                                                                                                                                                          >  1:149722/1‑301 (MQ=255)`  
                                                                                        `gAGGGCTTAGTATGTATCGAGAATAACTCATTCCGTGAGGCACGTAACTTGTTTGATTCGGACCTATCCCTCACTATGCCAGACGGACCGCAATTCTCAGTGAATGACCTAAGGGACTTCGATATGTTCCGCATCATGCCAGCGTATGACCGCCGTGTCAATGGTGACATCGCCATCATGGGGTCTACTGGTAAAACCACTAAGGAACTTAATGATGAGATTTTGGCTCTCAAAGCGAAAGCTGAGGGAGACGGTAAGAAGACTGGCGAGGTACATGCTTTAATGGATACCGGTAAGATTc                                                                                                                                                                                                                      <  1:135990/301‑1 (MQ=255)`  
                                                                                           `ggCTTAGTAGGTATCGAGAATAACTCATTCCTTGAGGCACGTAACTTGTTTGATTCGGACCTATCCATCACTATGCCAGACGGACAGCAATTCTCAGTGAATGACCTAAGGGACTTCGATATGTTCCGCATCATGCCAGCGTATGACCGCCGTGTCAATGGTTACATCGCCATCATGGGGTCTACTGGTAAAACCACTAAGGAACTTAAGGATGAGATTTTGGCTCTCAAAGCGAAAGCTGAGGGAGACGGTAAGAAGACTGGCGAGGTACATGCTTTAATGGATACCGTTAAGATTCttt                                                                                                                                                                                                                   >  1:104376/1‑300 (MQ=255)`  
                                                                                            `gCTTAGTAGGTATCGAGAATAACTCATTCCTTGAGGCACGTAACTTGTTTGATTCGGACCTATCCATCACTATGCCAGACGGACAGCAATTCTCAGTGAATGACCTAAGGGACTTCGATATGTTCCGCATCATGCCAGCGTATGACCGCCGTGTCAATGGTGACATCGCCATCATGGGGTCTACTGGTAAAACCACTAAGGAACTTAAGGATGAGATTTTGGCTCTCAAAGCGAAAGCTGAGGGAGACGGTAAGAAGACTGGCGAGGTACATGCTTTAATGGATACCGTTAAGATTCTTAc                                                                                                                                                                                                                  <  1:402061/301‑1 (MQ=255)`  
                                                                                            `ataagagacagaTCGAGAATAACTCATTACTTGAGGAACGTAACTTGTTTGATTCGGACCTATCCATCACTATGCCAGACGGACAGCAATTCTCAGTGAATGACCTAAGGGACTTCGATATGTTCCGCATCATGCCAGCGTATGACCATCGTGTCAATGGTTACATCGCCATCATGGGGTCTACTGGTAAAACCACTAAGGAACTTAAGGATGAGATTTTGGCTCTCAAAGCGAAAGCTGAGGGAGACGGTAAGAAGACTGGCGAGGTACATGCTTTAATGGATACCGTTAAGATTCTTAc                                                                                                                                                                                                                  <  1:298567/290‑1 (MQ=255)`  
                                                                                                 `gtagGTATCGAGAATAACTCATTCCTTGAGGCACGTAACTTGTTTGATTCGGACCTATCCATCACTATGCCAGACGGACAGCAATTCTCAGTGAACGACCTAAGGGACTTCGATATGTTCCGCATCATGCCAGCGTATGACCGCCGTGTCAATGGTGACATCGCCATCATGGGGTCTACTGGTAAAACCACTAAGGAACTTAATGATGAGATTTTGGCTCTCAAAGCGAAAGCTGAGGGAGACGGTAAGAAGACTGGCGAGGTACATGCTTTAATGGATACCGTTAAGATTCTTACTGGTc                                                                                                                                                                                                             >  1:190996/1‑301 (MQ=255)`  
                                                                                                    `ggTATCGAGAATAACTCATTCCTTGAGGCACGTAACTTGTTTGATTCGGACCTATCCATCACTATGCTAGACGGACAGCAATTCTCAGTGAATGACCTAAGGGACTTCGATATGTTCCGCATCATGCCAGCGTATGACCGCCGTGTCAATGGTGACATCGCCATCATGGGGTCTACTGGTAAAACCACTAAGGCACTTAAGGATGAGATTTTGGCTCTCAAAGCGAAAGCTGCGGGCGACGGTAAGAAGACTGGCGAGGTACATGCTTTAATGGATACCGTTAAGATTCTTACTGGTCGTg                                                                                                                                                                                                          >  1:304537/1‑301 (MQ=255)`  
                                                                                                      `agacaggaATAACTCATTCCTTGAGGCACGTAACTTGTTTTATTCGGACCTATCCATCACTATGCCAGACGGACAGCAATTCTCAGTGAATGACCTAAGGGACTTCGATATGTTCCGCATCATGCCAGCGTATGACCGCCGTGTCAATGGTGACATCGCCATCATGGGGTCTACTGGTAAAACCACTAAGGAACTTAAGGATGAGATTTTGGCTCTCAAAGCGAAAGCTGAGGGAGACGGTAAGAAGACTGGCGAGGTACATGCTTTAATGGATACCGTTAAGATTCTTACTGGTCGTGCt                                                                                                                                                                                                        <  1:269858/295‑1 (MQ=255)`  
                                                                                                       `gtggataagagacagttCCTTTAGGCACGTAACTTGTTTGATTCGGACCTATCCATCACTATGCCAGACGGAAAGCAATTCTCAGTGAATGACCTAAGGGACTTCGATATGTTCCGCATCATGCCAGCGTATGACCGCCGTGTCAATGGTTACATCGCCATCATGGGGTCTACTGGTAAAACCACTAAGGAACTTAAGGATGAGATTTTGGCTCTCAAAGCGAAAGCTGAGGGAGACGGTAAGAAGACTGGCGAGGTACATGCTTTAATGGATACCGTTAAGATTCTTACTGGTCGTGCTa                                                                                                                                                                                                       <  1:36774/286‑1 (MQ=255)`  
                                                                                                          `gagaATAACTCATTCCTTGAGGCACGTAACTTGTTTGATTCGGACCTATCCATCACTATGCCAGACGGACATCAATTCTCAGTGAATGACCTAAGGGACTTCGATATGTTCCGCATCATGCCAGCGTATGACCGCCGTGTCAATGGTGACATCGCCATCATGGGGTCTACTGGTAAAACCACTAAGGAACTTAAGGATGAGATTTTGGCTCTCAAAGCGAAAGCTGAGGGAGACGGTAAGAAGACTGGCGAGGTACATGCTTTAATGGATACCGTTAAGATTCTTAcctgtctcttataca                                                                                                                                                                                                    >  1:217141/1‑287 (MQ=255)`  
                                                                                                            `gaATAACTCATTCCTTGAGGCACGTAACTTGTTTGATTCGGACCTATCCATCACTATGCCAGACGGACAGCAATTCTCAGTGAATGACCTAAGGGACTTCGATATGTTCCGCATCATGCCAGCGTATGACCGCCGTGTCAATGGTTACATCGCCATAATGGGGTCTACTGGTAAAACCACTAAGGAACTTAAGGATGAGATTTTGGCTCTCAAAGCGAAAGCTGAGGGAGACGGTAAGAAGACTGGCGAGGTACATGCTTTAATGGATACCGTTAAGATTCTTACTGGTCGTGCTAGACGc                                                                                                                                                                                                  >  1:426154/1‑301 (MQ=255)`  
                                                                                                                `aaCTCATTCCTTGAGGCACGTAACTTGTTTGATTCGGACCTATCCATCACTATGCCAGACGGACAGCAATTCTCAGTGAATGACCTAAGGGACTTCGATATGTTCCGCATCATGCCAGCGTATGACCGCCGTGTCAATGGTTACATCTCCATCATGGGGTCTACTGGTTAAACCACTAAGGAACTTAAGGATGAGATTTTGGCTCTCAAAGCGAAAGATGAGGGAGACGGTAAGAAGACTGGCGAGGTACTTGCTTTAATGGATACCGTTAAGGTTCTTACCGGTCGTGCtctgtctcttt                                                                                                                                                                                              >  1:430085/1‑291 (MQ=255)`  
                                                                                                                     `ggtcggagatgtgtatgagagccccgATTCGGACCTATCCATCACTATGCCAGACTGACAGCAATTCTCAGTGCATGACCTACGGGACTTCGATATGTTCCCCATCATGCCAGCGTATGACCGCCGTGTCAATGGTGACATCGCTCTCATGGGTTCTTCTGGTAAAACCACTAAAAAACTTAAGGATTAGATTTTGGCTCTTAAAGCGAATGCTGTGGGAGACGGTAATTAGACTGGCGAGGTACATGCTTTAATTTATACCGTTAAGATTCTTACTGGTCGTGCTAGACGCAATCAGGac                                                                                                                                                                                         <  1:261341/276‑1 (MQ=255)`  
                                                                                                                         `cTTGAGGCACGTAACTTGTTTGATTCGGACCTATCCATCAATATGCCAGAAGGACAGCAATTCTCAGTGAATGACCTAAGGGACTTCGATATGTTCCGCATCATGCCAGCGTATGACCGCCGTGTCAATTGTGACATCGCCATCATGGGGTCTACTGGTAAAACCACTAAGGAACTTAAGGATGAGATTTTGGCTCTCAAAGCGAAAGCTGAGGGAGACGGTAAGAAGACTGGCGAGGTACATGCTTTAATGGATACCGTTAAGATTCTTACTGGTCGTGCTAGACGCAATCAGGACACtg                                                                                                                                                                                     <  1:363939/301‑1 (MQ=255)`  
                                                                                                                          `ttGAGGCACGTAACTTGTTTGATTCGGACCTATCCATCACTATGCCAGACGGACAGCAATTCTCAGTGAAGGAACTAAGGGACTTCGATATGTTCCGCATCATGCCAGCGTATGACCGCCGTGTCAATGGTGACATCGCCATCATGGGGTCTACTGGTAAAACCACTAAGGAACTTAAGGATGAGATTTTGGCTCTCAAAGCGAAAGCTGAGGGAGACGGTAAGAAGACTGGCGAGGTACATGCTTTAATGGATACCGTTAAGATTCTTACTGGTCGTGCTAGACGCAATCAGGACACtgt                                                                                                                                                                                    <  1:162917/301‑1 (MQ=255)`  
                                                                                                                               `gCACGTAACTTGTTTGATTCGGACCTATCCATCACTATGACAGACGGACAGCAATTCGCAGTGAATGACCTAAGGGACTTCGATATGTTCCGCATCATGCCAGCGTATGACCGCCGTGTCAATGGTTACATCGCCATCATGGGGTCTACTGGTAAAACCACTAAGGAACTTAAGGATGAGATTTTGGCTCTCAAAGCGAAAGCTGAGGGAGACGGTAAGAAGACTGGCGAGGTACATGCTTTAATGGATACCGTTAAGATTCTTACTGGTCGTGCTAGACGCAATCAGGACACTGTGTggg                                                                                                                                                                               <  1:226138/301‑1 (MQ=255)`  
                                                                                                                                 `ctcggagaggggtataagagacagaTCCATCAATATGCCAGACGGACAGCAATTCACAGTGAATGACCTAAGGGACTTCGATATGTTCCGCATCATGCCAGCGTATGACCGCCGTGTCAATGGTGACATCGCCATCATGGGGTCTACTGGTAAAACCACTAAGGAACTTAAGGATGAGATTTTGGCTCTCAAAGCGAAAGCTGAGGGAGACGGTAAGAAGACTGGCGAGGTACATGCTTTAATGGATACCGTTAAGATTCTTACTGGTCGTGCTAGACGCAATCAGGACACTGTGTGGGaa                                                                                                                                                                             <  1:341905/277‑1 (MQ=255)`  
                                                                                                                                 `agatgtgtataagagacaggACCTATCCATCACTATGCCAGATGGACAGCAATTCTCAGTGAATGACCTAAGGGACTTCGATATGTTCCGCATCATGCCAGCGTATGACCGCCGTGTCAATGGTGACATCGCCATCATGGGGTCTACTGGTAAAACCACTAAGGAACTTAAGGATGAGATTTTGGCTCTCAAAGCGAAAGCTGAGGGAGACGGTAAGAAGACTGGCGAGGTACATGCTTTAATGGATACCGTTAAGATTCTTACTGGTCGTGCTAGACGCAATCAGGACACTGTGTGGGaa                                                                                                                                                                             <  1:208527/283‑1 (MQ=255)`  
                                                                                                                                 `aCGTAACTTGTTTGATTCGGCCCTATCCATCACTATGCCAGACGGACAGCAATTCTCAGTGAATGACCTAAGGGACTTCGATATGTTCCGCATCATGCCAGCGTTTGACCGCCGTGTCAATGGTTACATCGCCATCATGGGGTCTACTGGTAAAACCACTAAGGAACTTAAGGATGAGATTTTGGCTCTAAAAGCGAAAGCTGAGGGAGACGGTAAGAAGACTGGCGAGGTACATGCTTTAATGGATACCGTTAAGATTCTTACTGGTCGTGCTAGACGCAATCAGGACACTGTGTGGGaa                                                                                                                                                                             <  1:243798/301‑1 (MQ=255)`  
                                                                                                                                          `gtgtataagagacagaTCCATCACTATGCCAGACGGACAGCAATTCTCAGTGAATGACCTAAGGGACTTCGATATGTTCCGCATCATGCCAGCGTATGACCGCCGTGTCAATGGTTACATCGCCATCATGGGGTCTACTGGTAAAACCACTAAGGAACTTAAGGATGAGATTTTGGCTCTCAAAGCGAAAGCTGAGGGAGACGGTAAGAAGACTGGCGAGGTACATGCTTTAATGGATACCGTTAAGATTCTTACTGGTCGTGCTAGACGCAATCAGGACACTGTGTGGGAAACCTCACTg                                                                                                                                                                    <  1:239665/286‑1 (MQ=255)`  
                                                                                                                                              `gATTCGGACCTATCCATCACTATGCCAGACGGACAGCAATTCTCAGTGAATGACCTAAGGGACTTCGATATGTTCCGCATCATGCCAGCGTATGACCGCCATGTCAATGGTGACATCGCCATCATGGGGTCTACTGGTAAAACCACTAAGGAACTTAAGGATGAGATTTTGGCTCTCAAAGCGAAAGCTGAGGGAGACGGTAAGAAGACTGGCGAGGTACATGCTTTAATGGATACCGTTAAGATTCTTACTGGTCGTGCTAGACGCAATCAGGACACTGTGTGGGAAACCTCACTGCGTg                                                                                                                                                                >  1:361041/1‑301 (MQ=255)`  
                                                                                                                                               `atgcggcacTATCCATCACGATGCCAGACGGACAGCAATTCTCAGTGAATGACCTAAGGGACTTCGATATGTTCCGCATCATGCCAGCGTATGACCGCCGTGTCAATGGTGACATCGCCATCATGGGGTCTACTGGTAAAACCACTAAGGAACTTAAGGATGAGATTTTGGCTCTCAAAGCGAAAGCTGAGGGAGACGGTAAGAAGACTGGCGAGGTACATGCTTTAATGGATACCGTTAAGATTCTTACTGGTCGTGCTAGACGCAATCAGGACACTGTGTGGGAAACCTCACTGCGTGc                                                                                                                                                               <  1:126747/293‑1 (MQ=255)`  
                                                                                                                                               `aTTCGGACCTATCCATCACTATGCCAGACGGACAGCAATTCTCAGTGAATGACCTAAGGGACTTCGATATGTTCCGCATCATGCCAGCGTATGACCGCCGTGTCAATGGTGACATCGCCATCATGGGGTCTACTGGTAAAACCACTAAGTAACTTAATGATGAGATTTTGGCTCTCAAAGCGAAAGCTGAGGGAGACGGTAAGAAGACTGGCGAGGTACATGCTTTAATGGATACCGTTAAGATTCTTACTGGTCGTGCTAGACGCAATCAGGACACTGTGTGGGAAACCTCACTGCGTGc                                                                                                                                                               >  1:49752/1‑301 (MQ=255)`  
                                                                                                                                               `aTTCGGACCTATCCATCACTATGCCAGACGGACAGCAATTCTCAGTGAATGACCTAAGGGACTTCGATATGTTCAGAATCATGCCAGCGTATGACCGCCGTGTCAATGGTTACATCGCCATCATGGGGTCTACTGGTAAAACCACTAAGGAACTTAAGAATGAGATTTTGGCTCTCAAAGCGAAAGCTGAGGGAGACGGTAAGAAGACTGGCGAGGTACATGCTTTAATGGATACCGTTAAGATTCTTACTGGTCGTGCTAGACGCAATCAGGACACTGTGTGGGAAACCTCACTGCGTGc                                                                                                                                                               >  1:502458/1‑301 (MQ=255)`  
                                                                                                                                                   `ggACCTATCCATCACTATGCCAGACGGACAGCAATTCTCAGTGAATGACCTAAGAGACTTCGATATGTTCCGCATCATGCCAGCGTATGACCGCCGTGTCAATGGTGACATCGCCATCATGGGGTCTACTGGTAAAACCACTAAGGAACTTAATGATGAGATTTTGGCTCTCAAAGCGAAAGCTGAGGGAGACGGTAAGAAGACTGGCGAGGTACATGCTTTAATGGATACCGTTAAGATTCTTACTGGTCGTGCTAGACGCAATCAGGACACTGTGTGGGAAACCCCACTGCGTGCCATc                                                                                                                                                           >  1:220741/1‑301 (MQ=255)`  
                                                                                                                                                         `aTCCATCACTATGCCAGACGGACAGCAATTCTCAGTGAATGACCTAAGGGACTTCGATATGTTCCGCATTATGCCAGCGTATGACCGCCGTGTCAATGGTGACATCGCCATCATGGGGTCTACTGGTAAAACCACTAAGGAACTTAATGATGAGATTTTGGCTCTCAAAGCGAAAGCTGAGGGAGACGGTAAGAAGACTGGCGAGGTACATGCTTTAATGGATACCGTTAAGATTCTTACTGGTCGTGCTAGACGCAATCAGGACACTGTGTGGGAAACCTCACTGCGTGCCATCAATGAc                                                                                                                                                     <  1:277728/301‑1 (MQ=255)`  
                                                                                                                                                         `aTCCATCACTATGCCAGACGGACAGCAATTCTCAGTGAATGACCTAAGGGACTTCGATATGTTCCGCATCATGCCAGCGTATGACCGCCGTGTCAATGGTTACATCGCCATCATGGGGTCTACTGGTAAAACCACTAAGGAACTTAAGGATGAGATTTTGGCTCTCAAAGCGAAAGCTGAGGGAGACGGTAAGAAGACTGGCGAGGTACATGCTTTAATGGATACCGTTAAGATTCTTACTGGTCGTGCTAGACGCAATCAGGACACTGTGTGGGAAACCTCACTGCGTGCCATCAATGAc                                                                                                                                                     >  1:392027/1‑301 (MQ=255)`  
                                                                                                                                                         `aTCCATCACTATGCCAGACGGACAGCAATTCTCAGTGAATGACCTAAGGGACTTCGATATGTTCCGCATCATGCCAGCGTATGACCGCCGTGTCAATGGTTACATCGCCATCATGGGGTCTACTGGTAAAACCACTAAGGAACTTAAGGATGAGATTTTGGCTCTCAAAGCGAAAGCTGAGGGAGACGGTAAGAAGACTGGCGAGGTACATGCTTTAATGGATACCGTTAAGATTCTTACTGGTCGTGCTAGACGCAATCAGGACACGGTGTGGGAAACCTCACTGCCTGTCacttatacc                                                                                                                                                     >  1:286881/1‑293 (MQ=255)`  
                                                                                                                                                         `aTCCATCACTATGCCAGACGGACAGCAATTCTCAGTGAATGACCTAAGGGACTTCGATATGTTCCGCATCATGCCAGCGTATGACCGCCGTGTCAATGGTGACATCGCCATCATGGGGTCTACTGGTAAAACCACTAAGGAACTTAAGGATGAGATTTTGGCTCTCAAAGCGAAAGCTGAGGGAGACGGTAAGAAGACTGGCGAGGTACATGCTTTAATGGATACCGTTAAGATTCTTACTGGTCGTGCTAGACGCAATCAGGACACTGTGTGGGCAACCTCAATGctgtctcttatacac                                                                                                                                                     >  1:314504/1‑287 (MQ=255)`  
                                                                                                                                                               `cACTATGCCAGACGGACAGCAATTTTCAGTGAATGACCTAAGGTACTTCGATATGTTCTGCATCATGCCAGCGTATGACCGCCGTGTCAATGGTTACATCGCCATCATGGGGTCTACTGGTAAAACCACTAAGGAACTTAAGGATGAGATTTTGGCTCTCAAAGCGAAAGCTGAGGGAGACGGTAAGAAGACTGGCGAGGTACATGCTTTAATGGATACCGTTAAGATTCTTACTGTTCGTGCTAGACGCAATCAGGACACTGTGTGGGAAACCTCACTGCGTGCCATCAATGACCTAggg                                                                                                                                               <  1:252131/301‑1 (MQ=255)`  
                                                                                                                                                                `aagaTGCCATACGGACAGCAATTCTCAGTGAATGACCTAAGGGACTTCGATATGTTCCGCATCATGCCAGCGTATGACCGCCGTGTCAATGGTGACATCGCCATCATGGGGTCTACTGGTAAAACAACTAAGGAACTTAAGGATGAGATTTTGGCTCTCAAAGCGAAAGCTGAGGGAGACGGTAAGAAGACTGGCGAGGTACATGCTTTAATGGATACCGTTAAGATTCTTACTGGTCGTGCTAGACGCAATCAGGACACTGTGTGGGAAACCTCACTGCGTGCCATCAATGACCTAGGGt                                                                                                                                              <  1:166579/298‑1 (MQ=255)`  
                                                                                                                                                                 `cTATGCCAGACGGACAGCAATTCTCAGTGAATGACCTAAGGGACTTCGATATGTTCCGCATCATGCCAGCGTATGACCGCCGTGTCAATGGTTACATCGCCATCATGGGGTCTACTGGTAAAACCACTAAGGAACTTAAGGATGCGATTTTGGCTCTCAAAGCGAAAGCTGAGGGAGACGGTAAGAAGACTGGCGAGGTACATGCTTTAATGGATACCGTTAAGATTCTTACTGGTCGTGCTAGACGCAATCAGGACACTGTGTGGGAAACCTCACTGCGTGCCATCAATGACCtgtctct                                                                                                                                             >  1:436337/1‑295 (MQ=255)`  
                                                                                                                                                                   `aTGCCAGACGGACAGCAATTCTCAGTGAATGACCTAAGGGACTTCGATATGTTCCGCATCATGCCAGCGTATGACCGCCGTGTCAATGGTGACATCGCCATCATGGGGTCTACTGGTAATACCACTAAGGAACTTAAGGATGAGATTTTGGCTCTCAAAGCGAAAGCTGAGGGAGACGGTAAGAAGACTGGCGAGGTACATGCTTTAATGGATACAGTTAAGATTCTTACTGGTCGTGCTAGACGCAATCAGGACACTGTGTGGGAAACCTCACTGCGTGCCATCAATGACCtctgtctct                                                                                                                                           >  1:95733/1‑293 (MQ=255)`  
                                                                                                                                                                     `gCCAGACGGACAGCAATTCTCAGTGAATGACCTAAGGGACTTCGATATGTTCCGCATGATGCCAGCGTATGACCGCCGTGTCAATGGTTACATCGCCATCATGGGGTCTACTGGTAAAACCACTAAGGAACTTAAGGATGAGATTTTGGCTCTCAAAGCGAAAGCTGAGGGAGACGGTAAGAAGACTGGCGAGGTACATGCTTTAATGTATACCGTTAAGATTCTTACTGGTCGTGCTAGACGCAATCAGGACACTGTGTGGGAAACCTCACTGCGTGCCATCAATGACCTAGGGttcttc                                                                                                                                         <  1:336677/301‑1 (MQ=255)`  
                                                                                                                                                                       `cAGACCGACAGCAATTCTCAGGGAATGACCTAAGGGACTTCGATATGTTCCGCATCATGCCAGCGTATGACCGCCGTGTCAATGGTGACATCGCCATCATGGGGTATACTGGTAAAACCACTAAGGAACTTAATGATGAGATTTTGGCTCTCAAAGCGAAAGCTGAGGGAGACGGTAAGAAGACTGGCGAGGTACATGCTTTAATGGATACCGTTAAGATTCTTACTGGTCGTGCTAGACGCAATCAGGACACTGTGTGGGAAACCTCACTGCGTGCCATCAATGACCTAGGGTTCTTCGc                                                                                                                                       <  1:440968/301‑1 (MQ=255)`  
                                                                                                                                                                               `cAGCAATTCTCAGTGAATGACCTAAGGGACTTCGATATGTTCCGCATCATGCCAGCGTATGACCGCCGTGTCAATGGTGACATCGCCATCATGGGGTCTACTGGTAAAACCACTAAGGAACTTAATGATTAGATTTTGGCTCTCAAAGCTAAAGCTGAGGGAGACGGTAAGAAGACTGGCGAGGTACATGCTTTAATGGATACCGTAAAGATTCTTACTGGTCGTGCTAGCCGCAATCAGGACACTGTGTGGGAAACCTCACTGCGTGCCATCAATGACCTAGGGTTCTTCGCTACGAACg                                                                                                                               >  1:181049/1‑301 (MQ=255)`  
                                                                                                                                                                                    `aTTCTCAGTGAATGACCTAAGGGACTTCGATATGTTCCGCATCATGCCAGCGTATGACCGACGTGTCAATGGTGACATCGCCATCATGGGGTCTACTGGTAAAACCACTAAGGAACTTAATTATGAGATTTTGGCTCTCAAAGCGAAAGCTGAGGGAGACGGTAAGAAGACTGTCGAGGTACATGCTTTAATGGATACCGTTAAGATTCTTACTGGTCGTGCTAGACGCAATCAGGACACTGTGTGGGAAACCTCACTGCGTGCCATCAATGACCTAGGGTTCTTCGCTAAGAACGCCTAc                                                                                                                          <  1:81949/301‑1 (MQ=255)`  
                                                                                                                                                                                      `tctcAGTGAATGACCTAAGGGAATTCGATATGTTCCGCATCATGCCAGCGTATGACCGCCGTGTCAATGGTTACATCGCCATCATGGGGTCTACTGGTAAAACCACTAAGGAACTTAAGGATGAGATTTTGGCTCTCAAAGCGAAAGCTGAGGGAGACGGTAAGAAGACTGGCGAGGTACATGCTTTAATGGATACCGTTAAGATTCTTACTGGTCGTGCTAGACGCAATCAGGACACTGTGTGGGAAACCTCACTGCGTGCCATCAATGACCTAGGGTTCTTCGCTAAGAACGCCTACAt                                                                                                                        <  1:87476/301‑1 (MQ=255)`  
                                                                                                                                                                                      `gacaggTGAATGACCTAAGGGACTTCGATATGTTCCGCATCATGCCAGCGTATGACCGCCGTGTCAATGGTTACATCGCCATCATGGGGTCTACTGGTAAAACCACTAAGGAACTTAAGGATGAGATTTTGGCTCTCAAAGCGAAAGCTGAGGGAGACGGTAAGAAGACTGGCGAGGTACATGCTTTAATGGATACCGTTAAGATTCTTACTGGTCGTGCTAGACGCAATCAGGACACTGTGTGGGAAACCTCACTGCGTGCCATCAATGACCTAGGGTTCTTCGCTAAGAACGCCTACAt                                                                                                                        <  1:6063/296‑1 (MQ=255)`  
                                                                                                                                                                                         `cAGTGAATGACCTAAGGGACTTCGATATGTTCCGCATCATGCCAGCGTATGACCGCCGTGTCAATGGTTACATCGCCATCATGGGGTCTACTGGTAAAACCACTAAGGAACTTAATGATGAGATTTTGGCTCTCAAAGCGAAAGCTGAGGGAGACGGTAAGAAGACTGGCGAGGTACATGCTTTAATGGATACCGTTAAGATTCTTACTGGTCGTGCTAGACGCAATCAGGACACTGTGTGGTAAACCTCACTGCGTGCCATCAATGACCTAGGGTTCTTCGCTAAGAACGCCTACATggg                                                                                                                     <  1:71448/301‑1 (MQ=255)`  
                                                                                                                                                                                           `gTGAATGACCTAAGGGACTTCGATATGTTCCGCATCATGCCAGCGTATGACCGCCGTGTCAATGGTTACATCGCCATCATGGGGTCTACTGGTAAAACCACTAAGGAACTTAAGGATGAGATTTTGGCTCTCAAAGCGAAAGCTGAGGGAGACGGTAAGAAGACTGGCGAGGTACATGCTTTAATGGATACCGTTAATATTCTTACTGGTCGTGCTAGACGCAATCAGGACACTGTGTGGGAAACCTCACTGCGTGCCATCAATGACCTAGGGTTCTTCGCTAAGAACGCCTACATgggct                                                                                                                   >  1:463460/1‑299 (MQ=255)`  
                                                                                                                                                                                           `gTGAATGACCTAAGGGACTTCGATATGTTCCGCATCATGCCAGCGTATGACCGCCGTGTCAATGGTTACATCGCCATCATGGGGTCTACTGGTAAAACCACTAAGGAACTTAAGGATGAGATTTTGGCTCTCAAAGCGAAAGCTGAGGGAGACGGTAAGAAGACTGGCGAGGTACATGCTTTAATGGATACCGTTAAGATTCTTACTGGTCGTGCTAGACGCAATCAGGACAATGTGTGGGAAACCTCACTGAGTGCCATAAATGACCTAGGGttattcctgtctattatcaacatctccg                                                                                                                   >  1:154225/1‑279 (MQ=255)`  
                                                                                                                                                                                                  `aCCTAAGGGACTTCGATATGTTCCGCATCATGCCAGCGTATGACCGCCGTGTCAATGGTTACATCGCCATCATGGGGTCTACTGGTAAAACCACTAAGGAACTTAAGGATGAGATTTTGGCTCTCAAAGCGAAAGCTGAGGGAGACGGTAAGAAGACTGGCGAGGTACATGCTTTAATGGATACCGTTAAGATTCTTACTGGTCGTGCTAGACGCAATCAGGACACTGTGTGGGAAACCTCACTGCGTGCCATCAATGACCTAGGGTTCTTCGCTAAGAACGCCTACATGGGTGCTCAGaa                                                                                                            >  1:114509/1‑301 (MQ=255)`  
                                                                                                                                                                                                  `aCCTAAGGGACTTCGATATGTTCCGCATCATGCCAGCGTATGACCGCCGTGTCAATGGTTACATCGCCATCATGGGGTCTACTGGTAAAACCACTAAGGAACTTAAGGATGAGATTTTGGCTCTCAAAGCGAAAGCTAAGGGAGACGGTAAGAAGACTGGCGAGGTACATGCTTTAATGGATACCGTTAAGATTCTTACTGGTCGTGCTAGACGCAATCAGGACACTGTGTGGGAAACCTCACTGCGTGCCATCAATGACCTAGGGTTCTTCGCTAAGAACGCCTACCTgtctcttattca                                                                                                            >  1:230709/1‑290 (MQ=255)`  
                                                                                                                                                                                                  `aCCTAAGGGACTTCGATATGTTCCGCATCATGCCAGCGTATGACCGCCGTGTCAATGGTTACATCGCCATCATGGGGTCTACTGGTAAAACCACTAAGGAACTTAAGGATGAGATTTTGGCTCTCAAAGCGAAAGCTAAGGGAGACGGTAAGAAGACTGGCGAGGTACATGCTTTAATGGATACCGTTAAGATTCTTACTGGTCGTGCTAGACGCAATCAGGACACTGTGTGGGAAACCTCACTGCGTGCCATCAATGACCTAGGGTTCTTCGCTAAGAACGCCTACAtctgtctcttata                                                                                                            >  1:234835/1‑289 (MQ=255)`  
                                                                                                                                                                                                  `aCCTAAGGGACTTCGATATGTTCCGCATCATGCCAGCGTATGACCGCCGTGTCAATGGTGACATCGCCATCATGGGGTCTACTGGTAAAACCACTAAGGAACTTAATGATGAGATTTTGGCTCTCAAAGCGAAAGCTGAGGGAGACGGTAAGAAGACTGGCGAGGTACATGCTTTAATGGATACCGTTAAGATTCTTACTGGTCGTGCTAGACGCAATCAGGACACTGGGTGGGAAACCTCACTGCGTGCCATCAATGACCTAGGGTTCTTCGCTAAGAACGCCTACATGGGTGCTAAGaa                                                                                                            >  1:37780/1‑301 (MQ=255)`  
                                                                                                                                                                                                  `aCCTAAGGGACTTCGATATGTTCCGCATCATGCCAGCGTATGACCGCCGTGTCAATGGTGACATCGCCATCATGGGGTCTACTGGTAAAACCACTAAGGAACTTAAGGATGAGATTTTGGCTCTCAAAGCGAAAGCTGAGGGAGACGGTAAGAAGACTGGCGAGGTACATGCTTTAATGGATACCGTTAAGATTCTTACTGGTCGTGCTAGACGCAATCAGGACACTGTGTGGGAAACCTCACTGCGTGCCATCAATGACCTAGGGTTCTTCGCTAAGAACGCCTACATGGGTGCTCAGaa                                                                                                            >  1:29187/1‑301 (MQ=255)`  
                                                                                                                                                                                                   `ccTAAGGGCCTTCGATATGTTCCGCATCATGCCAGCGTATGACCGCCGTGTCAATGGTTACATCGCCATCATGGGGTCTACTGGTAAAACCACTAAGGAACTTAAGGATGAGATTTTGGCTCTCAAAGCGAAAGCTGAGGGAGACGGTAAGAAGACTGGCGAGGTACATGCTTTAATGGATACCGTTAAGCTTCTTACTGGTCGTGCTAGACGCAATCAGGACACTGTGTGGGAAACCTCACTGCGTGCCATCAATGACCTAGGGTTCTTCGCTAAGAACGCCTACATGGTTGCTCAGAAc                                                                                                           >  1:118856/1‑301 (MQ=255)`  
                                                                                                                                                                                                   `ccTAAGGGACTTCGATATGTTCCGCATCATGCCAGCGTATGACCGCCGTGTCAATGGTTACATCGCCATCATGGGGTCTACTGGTAAAACCACTAAGGAACTTAAGGATGAGATTTTGGCTCTCAAAGCGAAAGCTGAGGGAGACGGTAAGAAGACTGGCGAGGTACATGCTTTAATGGATACCGTTAAGATTCTTACTGGTCGTGCTAGACGCAATCAGGACACTGTGTGGGAAACCTCACTGCGTGCCATCAATGACCTAGGGTTATTCGCTAAGAACGCCTACATGGGTGCTCAGAAc                                                                                                           >  1:274370/1‑301 (MQ=255)`  
                                                                                                                                                                                                            `cTTCGATATGTTCCGCATCATGCCAGCGTATGACCGCCGTGTCAATGGTGACATCGCCATCATGGGGTCTACTGGTAAAACCACTAAGGAACTTAAGGATGAGATTTTGGCTCTCAAAGCGAAAGCTGATGGAGACGGTAAGAAGACTGGCGAGGTACATGCTTTAATGGATACCGTTAAGATTCTTACTGGTCGTGCTAGACGCAATCAGGACACTGTGTGGGAAACCTCACTGCGTGCCATCAATGACCTAGGGTTCTTCGCTAAGAACGCCTACATGGGTGCTCAGAACATTACGgag                                                                                                  <  1:450765/301‑1 (MQ=255)`  
                                                                                                                                                                                                                     `gTTCCGCATCATGCCAGCGTATGACCGCCGTGTCAATGGTGACATCGCCATCATGGGGTCTACTGGTAAAACCACTAAGGAACTTAATGATGAGATTTTGGCTCTCAAAGCGAAAGCTGAGGGAGACGGTAAGAAGACTGGCGAGGTACATGCTTTAATGGATACCGTTAAGATTCTTACTGGTCGTGCTAGACGCAATCAGGACACTGTGTGGGAAACCTCACTGCGTGCCATCAATTACCTAGGGTTCTTCGCTAAGACCGCCTACATGGGTGCTCAGAACATTACGGATATTGCTggg                                                                                         >  1:422862/1‑301 (MQ=255)`  
                                                                                                                                                                                                                           `catcatGCCAGCGTATGACCGCCGTGTCAATGGTGACATCGCCATCATGGGGTCTACTGGTAAAACCACTAAGGAACTTAATGATGAGATTTTGGCTCTCAAAGCGAAAGCTGAGGGAGACGGTAAGAAGACTGTCGAGGTACATGCTTTAATGGATACCGTTAAGATTCTTACTGGTCGTGCTAGACGCAATCAGGACACTGTGTGGGAAACCTCACTGCGTGCCATCAATGACCTAGGGTTCTTCGCTCAGAACGCCTACATGGGTGCTCAGAACATTACGGAGACTgtctcttatacc                                                                                   >  1:140530/1‑290 (MQ=255)`  
                                                                                                                                                                                                                                `tGCCAGCGTATGACCGCCGTGTAAATGGTGACATCGCCATCATGGGGTCTACTGGTAAAACCACTAAGGAACTTAATGATGAGATTTTGGCTCTCAAAGCGAAAGCTGAGGGAGACGGTAAGAAGACTGGCGAGGTACATGCTTTAATGGATACCGTTAAGATTCTTACTGGTCGTGCTAGACGCAATCAGGACACTGTGTGGGAAACCTCACTGTGTGCCATCAATGACCTAGGGTTCTTCGCTAAGAACGCCTACATGGGTGCTCAGAACATTACGGAGATTGCTGGGATGATTGTCAc                                                                              <  1:195664/301‑1 (MQ=255)`  
                                                                                                                                                                                                                                     `gCGTATGACCGCCGTGTCAATGGTTACATCGCCATCATGGGGTCTACTGGTAAAACCACTAAGGAACTTAAGGATGAGATTTTGGCTCTCAAAGCGAAAGCTGAGGGAGACGGTAAGAAGACTGGCGAGGTACATGCTTTAATGGATACCGTTAAGATTCTTACTGGTCGTGCTAGACGCAATCAGGACACTGTGTGGGAAACCTCACTACGTGCCATCAATGACCTAGGGTTCTTCGCTAAGCACGCCTACATGGGTGCTCAGAACATTACGGAGATTGCTGGGACGATTGTCAATGGTa                                                                         >  1:45646/1‑301 (MQ=255)`  
                                                                                                                                                                                                                                      `cGTATGACCGCCGTGTCAATGGTGACATCGCCATCATGGGGTCTACTGGTAAAACCACTAAGGAACTTAATGATGAGATTTTGGCTCTCAAAGCGAAAGCTGAGGGAGACTGTAAGAAGACTGGCGAGGTACATGCTTTAATGGATACCGTTAAGATTCTTACTGGTCGTGCTAGACGCAATCAGGACACTGTGTGGGAAACCTCACGGCGTGCCATCAATGACCTAGGGTTCTTCGCTAAGAACGCCTACATGGGTGCTCAGAACATTACGGAGATTGCTGGGATGATTGTCACTGGTaa                                                                        <  1:186020/301‑1 (MQ=255)`  
                                                                                                                                                                                                                                        `ataagagacagtgtCAATGGTGACATCGCCATCATGGGGTCTACTGGTAAAACCACTAAGGAACTTAAGGATGAGATTTTGGCTCTCAAAGCGAAAGCTGAGGGAGACGGTAAGAAGACTGGCGAGGTACATTCTTTAATGGATACCGTTAAGATTCTTACTGGTCGTGCTAGACGCAATCAGGACACTGTGTGGGAAACCTCACTGCGTGCCATCAATGACCTAGGGTTCTTCGCTAAGAACGCCTACATTGGTGCTCAGAACATTACGGAGATTGCTGGGATGATTGTCACTGGTAACg                                                                      <  1:265329/291‑1 (MQ=255)`  
                                                                                                                                                                                                                                          `tGACCGCCGTGTCAATGGTGACATCGCCATCATGGGGTCTACTGGTAAAACCACTAAGGAACTTAAGGATGAGATTTTGGCTCTCAAAGCGAAAGCTGAGGGAGACGGTAAGAAGACTGGCGAGGTACATGCTTTAATGGATACCGTTAAGATTCTTACTGGTCGTGCTAGACGCAATCAGGACACTGTGTGGGAAACCTCACTGCGTGCCATCAATGACCTAGGGTTCTTCGCTAAGAACGCCTACATGGGTGCTCAGAACATTACGGAGATTGCTGGGATGATTGTCACTGGTAACgag                                                                    <  1:445329/301‑3 (MQ=255)`  
                                                                                                                                                                                                                                               `tccgTGTCAATGGTTACATCGCCATCATTGGGTCTACTGGTAAAACCACTAATGAACTTAAGGATGAGATTTTGGCTCTCAAAGCGAAAGCTGAGGGAGACGGTAAGAAGACTGGCGAGGTACATGCTTTAATGGATACCGTTAAGATTCTTACTGGTCGTGCTAGACGCAATCAGGACACTGTGTGGGAAACCTCACTGCGTGCCATCAATGACCTATGGTTCTTCGCTAAGAACGCCTACATGGGTGCTCAGAACATTACGGAGATTGCTGGGATGATTGTCACTGGTAACGTTCGTGc                                                               <  1:131454/300‑1 (MQ=255)`  
                                                                                                                                                                                                                                                  `gtgtCAATGGGGACATCGCCATCATGGGGTCTACTGGTAAAACCACTAAGGAACTTAAGGATGAGATTTTGGCTCTCAAAGCGAAAGCTGAGGGAGACGGTAAGAAGACTGGCGAGGTACATGCTTTAATGGATACCGTTAAGATTCTTACTGGTCGTGCTAGACGCAATCAGGACACTGTGTGGCAAACCTCACTGCGTGCCATCAATGACCTAGGGTTCTTCGCTAAGAACGCCTACATGGGTGCTCAGAACATTACGGAGATTGCTGGGATGATTGTCACTGGTAACGTTCGTGctct                                                            <  1:382951/301‑1 (MQ=255)`  
                                                                                                                                                                                                                                                    `gtCAATGGTGACATCGCCATCATGGGGTCTACTGGTAAAACCACTAAGGAACTTAAGGATGAGAATTTGGCTCTCAAAGCGAAAGCTGAGGGAGACGGTAAGAAGACTGGCGAGGTACATGCTTTAATGGATACCGTTAAGATTCTTACTGGTCGTGCTAGACGCAATCAGGACACTGTGTGGGAAACCTCACTGCGTGCCATCAATGACCTAGGGTTCTTCGCTAAGAACGCCTACATGGGTGCTCAGAACATTACGGAGATTGCTGGGATGATTGTCACTGGTAACGTTCGTGCTCTAg                                                          >  1:485104/1‑301 (MQ=255)`  
                                                                                                                                                                                                                                                       `ttataagagacagcaatcatGGGGTCTACTGGTAAAACCACTAAGGAAGTTAAGGATGAGCTTGTGTCTCTCAAAGCGAAAGCTGAGGGAGACGGTAAGAAGACTGGCGAGGTACATGCTGTAATGGATACAGTTAAGATTCTTACTGGTCGTGCTAGACGCAATCAGGACACTGTGTTGGAAACCTCACTGCGTGCCATCAATGACCTAGGGTTCTTCGCTAAGAACGCCTACATGGGTGCTAAGAACATTACGGAGATTGCTGGGATGATTGTCATTGGTAACGTTCGTGCTCTAGGGc                                                       <  1:309940/286‑1 (MQ=255)`  
                                                                                                                                                                                                                                                       `aaTGGTTACATCGCCATCATGGGGTCTACTGGTAAAACCACTAAGGAACTTAAGGATGAGATTTTGGCTCTCAAAGCGAAAGCTGAGGGAGACGGTAAGAAGACTGGCGAGGTACATGCTTTAATGGATACCGTTAAGATTCTTACTGGTCGTGCTAGACGCAATCAGGACACTGTGTGGGAAACCTAACTGCGTGCCATCAATGACCTAGGGTTCTTCGCTAAGAACGCCTACATGGGTGCTCAGAACATTACGGAGATTGCTGGGATGATTGTCACTGGTAACGTTCGTGCTCTAGGGc                                                       <  1:415156/301‑1 (MQ=255)`  
                                                                                                                                                                                                                                                         `tGGTTACATCGCCATAAGGGGGTCTACTGGTAAAACCACTAAGGAACTTAAGGATGAGATTTTGGCTCTCAAAGCGAAAGCTGAGGGAGACGGTAAGAAGACTGGCGAGGTACATGCTTTAATGGATACCGTTAAGATTCTTACTGGTCGTGCTAGACGCAATCAGTACACTGTGTGGGAAACCTCACTGCGTGCCATCAATGACCTAGGGTTCTTCGCTAAGAACGCCTACATGGGTGCTCAGAACATTACGGAGATTGCTGGGATGATTGTCACTGGTAACGTTCGTGCTCTAGGGCAt                                                     <  1:75998/301‑1 (MQ=255)`  
                                                                                                                                                                                                                                                           `gTGACATCGCCAACATGGGGTCTAATGGTAAAACCACTAAGGAACTTAAGGATGAGATTGTGGCTATGAAAGCGAAAGCTGAGGGAGACGGTAAGAAGACTGGCGAGGTACATGCTTTAATGGATACCGTTAAGATTCTTACTGGTCGTGCTAGACGCAATCAGGACACTGTGTGGGAAACCTCACTGCGTGCCATCAATGACCTAGGGTTCTTCGCTAAGAACGCCTACATGGGTGCTCAGAACATTACGGAGATTGCTGGGATGATTGTCACTGGTAACGTTCGTGCTCTAGGGCATgg                                                   <  1:109499/301‑1 (MQ=255)`  
                                                                                                                                                                                                                                                             `taCATCGCCATCATGGGATCTACTGGTAAAACCACGAAGGAACTTAAGGATGAGAGTTTGGCTCGCAAAGCGAAAGCTGAGGGAGACGGTAAGAAGACGGGCGAGGTACATGCTTGAATGGATACCGTTAAGATTCTTACTGGTCGTGCTAGACGCAATCAGGACACTGTGTGGGAAACCTCACTGCGTGCCATCAATGACCTAGGGTTCTTCGCTAAGAACGCCAACATGGGTGCTCAGAACATTACGGAGATTGCTTGGATGATTGTCACTGGTAACGTTCGTGCTCTAGGGCATGGTa                                                 <  1:176406/300‑1 (MQ=255)`  
                                                                                                                                                                                                                                                                                   `cTGGTAAAACCACTAAGGAACTTAAGGATGAGATTTTGGCTCTCAAAGCGAAAGCTGAGGGAGACGGTAAGAAGACTGGCGAGGTACATTCTTTAATGGATACCGTTAAGATTCTTACTGGTCGTGCTAGACGCAATCATGACACTGTGTGGGAAACCTCACTGCGTGCCATCAATGACCTAGGGTTCTTCGCTAAGAACGCCTACATGGGTGCTCAGAACATTACGGAGATTGCTGGGATGATTGTCACTGGTCACGTTCGTGCTCTAGGGCATGGTATCCCAATTCTGCgctgtctctt                           >  1:491295/1‑292 (MQ=255)`  
                                                                                                                                                                                                                                                                                     `ggTAAAACCACTAAGGAACTTAAGGATGAGATTTTGGCTCTCAAAGCGAACGCTGAGGGAGACGGTAAGAAGACTGGCGAGGTACATGCTTTAATGGATACCGTTAAGATTCTTACTGGTCGTGCTAGACGCAATCAGGACACTGTGTGGGAAACCTCACTGCGTGCCATCAATGACCTAGGGTTATTCGCTAAGAACGCCTACATGGGTGCTCAGAACATTACGGAGATTGCTGGGATGATTGTCACTGGTAACGTTCGTGCTCTAGGGCATGGTATCCCAATTcctgtctcttgtacaa                         >  1:248401/1‑286 (MQ=255)`  
                                                                                                                                                                                                                                                                                     `ggTAAAACCACTAAGGAACTTAAGGATGAGATTTTGGCTCTCAAAGCGAAAGCTGAGGGAGACGGTAAGAAGACTGGCGAGGTACATGCTTTAATGGATACCGTTAAGATTCTTACTGGTCGTGCTAGACGCAATCAGGACACTGTGTGGGAAACCTCACTGCGTGCCATCAATGACCTAGGGTTCTTCGCTAAGAACGCCTACATGGGTGCTCAGAACATTACGGAGATTGCTGGTATGATTGTCACTGGTAACGTTCGTGCTCTAGGGCATGGTATCCCAATTCTGCGTGATacctgtc                         >  1:356305/1‑296 (MQ=255)`  
                                                                                                                                                                                                                                                                                       `aaaaaCCACTAAGGAACGTAAGGATGGGGTTTTGGCACTCAAAGCGAAAGCTGAGGGAGACGGTAAGAAGACTGGCGAGGTACATGCTTTAATGGATACCGTTAAGATTCTTACTGGTCGTGCTAGACGCAATCAGGACACTGTGTGGGAAACCTCACTGCGTGCCATCAATGACCTAGGGTTCTTCGCTAAGAACGCCTACATGGGTGCTCAGAACATTACGGAGATTGCTGGGATGATTGTCACTGGTAACGTTCGTGCTCTAGGGCATGGTATCCCAATTCTGCGTGATACACTCTAc                       <  1:460190/300‑1 (MQ=255)`  
                                                                                                                                                                                                                                                                                               `cTAAGGAACTTAAGGATGAGATTTTTGCTCTCAAAGCGAAAACTGAGGGAGACGGTAAGAAGACTGGCGAGGTACATGCTTTAATGGATACCGTTAAGATTCTTACTGGTCGTGCTAGACGCAATCAGGACACTGTGTGGGAAACCTCACTGCGTGCCATCAATGACCTAGGGTTCTTCGCTAAGAACGCCTACATGGGTGCTCAGAACATTACGGAGATTGCTGGGATGATTGTCACTGGTAACGTTCGTGCTCTAGGGCATGGTATCCCAATTCTGCGTGATACACTCTACactgtctc               >  1:328611/1‑294 (MQ=255)`  
                                                                                                                                                                                                                                                                                               `cTAAGGAACTTAAGGATGAGATTTTGGCTCTCAAAGCGAAAGCTGAGGGAGACGGTAAGAAGACTGGCGAGGTACATGCTTTAATGGATACCGTTAAGATTCTTACTGGTCGTGCTAGACGCAATCAGGACACTGTGTGGGAAACCTCACTGCGTGCCATCAATGACCTAGGGTTCTTCGCTAAGAACGCCTACATGGGTGCTCAGAACATTACGGAGATTGCTGGGATGATTGTCACTGGTAACGTTCGTGCTCTAGGGCATGGTATCCCAATTCTGCGTGATACAatctctgtctcttt               >  1:212066/1‑291 (MQ=255)`  
                                                                                                                                                                                                                                                                                                  `gggAACTTAAGGATGAGATTTTGGCTCTCAAAGCGAAAGCTGAGGGAGACGGTAAGAAGACTGGCGAGGTACATGCTTTAATGGATACCGTTAAGATTCTTACTGGTCGTGCTAGACGCAATCAGGACACTGTGTGGGAAACCTCACTGCGTGCCATCAATGACCTAGGGTTCTTCGCTAAGAACGCCTACATGGGTGCTCAGAACATTACGGAGATTGCTGGGATGATTGTCACTGGTAACGTTCGTGCTCTAGGGCATGGTATCCCAATTCTGCGTGATACACTCTACAAGTCTAAAcc            <  1:406317/300‑1 (MQ=255)`  

                                                                                                                                                                                                                                                                                                            `|`                                                                                                                                                                                                                                                                                                              
`GATGGTAGAGAAGTACGCTATGGATAAGGCTTATGGTATCTCCCACTCAGACCAGTTCACCAACAGTTCCATAATAGAAGAGAACATTGAGGGCTTAGTAGGTATCGAGAATAACTCATTCCTTGAGGCACGTAACTTGTTTGATTCGGACCTATCCATCACTATGCCAGACGGACAGCAATTCTCAGTGAATGACCTAAGGGACTTCGATATGTTCCGCATCATGCCAGCGTATGACCGCCGTGTCAATGGTGACATCGCCATCATGGGGTCTACTGGTAAAACCACTAAGGAACTTAAGGATGAGATTTTGGCTCTCAAAGCGAAAGCTGAGGGAGACGGTAAGAAGACTGGCGAGGTACATGCTTTAATGGATACCGTTAAGATTCTTACTGGTCGTGCTAGACGCAATCAGGACACTGTGTGGGAAACCTCACTGCGTGCCATCAATGACCTAGGGTTCTTCGCTAAGAACGCCTACATGGGTGCTCAGAACATTACGGAGATTGCTGGGATGATTGTCACTGGTAACGTTCGTGCTCTAGGGCATGGTATCCCAATTCTGCGTGATACACTCTACAAGTCTAAACCAGTTTCAGCT  >  NC_001604.1/32769‑33369`

  

Alignment Legend

Aligned base mismatch/match (shaded by quality score): `ATCG/ATCG` `< 3 ≤` `ATCG/ATCG` `< 10 ≤` `ATCG/ATCG` `< 22 ≤` `ATCG/ATCG` `< 34 ≤` `ATCG/ATCG` `< 38 ≤` `ATCG/ATCG`

Unaligned base: `atcg`    Masked matching base: `atcg`    Alignment gap: `‑`    Deleted base: `‑`
